# A qualitative exploration of the barriers and facilitators to the implementation of the alcohol assertive outreach model

**DOI:** 10.1093/alcalc/agaf003

**Published:** 2025-02-03

**Authors:** Laura H Scoles, Nikolaos Mylonas, Aansha Priyam, Stephen Blood, Amy O’Donnell, Colin Drummond, Karina Lovell, Stephen J Kaar

**Affiliations:** Division of Psychology and Mental Health, School of Health Sciences, Faculty of Biology, Medicine, and Health, The University of Manchester, Hathersage Road, Manchester M13 9WL, UK; Division of Psychology and Mental Health, School of Health Sciences, Faculty of Biology, Medicine, and Health, The University of Manchester, Hathersage Road, Manchester M13 9WL, UK; Addictions Research Team, Greater Manchester Mental Health NHS Foundation Trust, Addictions Services, Bury New Road, Prestwich, Manchester M25 3BL, UK; Addictions Research Team, Greater Manchester Mental Health NHS Foundation Trust, Addictions Services, Bury New Road, Prestwich, Manchester M25 3BL, UK; Addictions Research Team, Greater Manchester Mental Health NHS Foundation Trust, Addictions Services, Bury New Road, Prestwich, Manchester M25 3BL, UK; Population Health Sciences Institute, Faculty of Medical Science, Newcastle University, Baddiley-Clark Building, Richardson Road, Newcastle NE2 4AX, UK; Department of Addiction, Institute of Psychiatry, Psychology and Neuroscience, Kings College London, London WC2R 2LS, UK; Division of Nursing, Midwifery & Social Work, School of Health Sciences, Faculty of Biology, Medicine, and Health, The University of Manchester, Hathersage Road, Manchester M13 9WL, UK; Division of Psychology and Mental Health, School of Health Sciences, Faculty of Biology, Medicine, and Health, The University of Manchester, Hathersage Road, Manchester M13 9WL, UK; Addictions Research Team, Greater Manchester Mental Health NHS Foundation Trust, Addictions Services, Bury New Road, Prestwich, Manchester M25 3BL, UK

**Keywords:** qualitative, implementation science, alcohol assertive outreach, alcohol dependence, CFIR

## Abstract

Alcohol use disorder has adverse implications for individuals' health, utilisation of healthcare services, and societal costs. There are a group of individuals who frequently attend hospital for alcohol-related issues, have complex co-morbid needs, and experience barriers to engaging with specialised alcohol treatment services. To support these individuals and reduce healthcare system costs, Alcohol Assertive Outreach Treatment (AAOT) has been recommended. However, AAOT is not routinely used in the UK. Understanding the determinants of the implementation of AAOT can increase its utilisation and effectiveness. This study therefore employed the Consolidated Framework for Implementation Research (CFIR) framework to highlight barriers and facilitators to the successful and sustainable implementation of AAOT. Semi-structured interviews were conducted with twenty AAOT team staff members (team managers and outreach workers) from two North West England AAOT teams. Twenty-eight stakeholders (clinicians, commissioners, policy makers and academics across England) were also interviewed, who were considered to be key contributors to AAOT implementation, both within and external to North West England. Framework analysis based on the CFIR was conducted, whilst allowing for inductive coding where appropriate. Overall, participants recognised AAOT as acceptable and beneficial. Three main themes were identified: organisational and individual level factors, including team culture and staff characteristics; systemic partnerships and interagency communication; and an adaptable model driven by research and evaluation. Each theme relates to various CFIR domains and constructs which were perceived to influence the implementation of AAOT. Readers are encouraged to consider the findings in the development and implementation of AAOT teams, new or existing.

## Teaser

**Short Summary:** Alcohol Assertive Outreach Treatment (AAOT) is an effective intervention for supporting individuals with alcohol dependence who may experience barriers to engaging with specialist alcohol treatment services. This qualitative study explored determinants to the successful and sustainable implementation of AAOT. Findings can support the development of AAOT services, new and existing.

## Introduction

Alcohol use disorders are associated with excess morbidity and mortality, including from liver cirrhosis, cancers, and mental health difficulties (Anderson et al. 2023). Alcohol-related hospital admissions are increasing in England, with estimates of 280,000 admissions in 2019/2020 (NHS England 2022). There has been a steep rise in alcohol-related deaths in England, which at 16 per 100,000 in 2022 (10,048 deaths), were the highest on record, up 32.8% since pre-pandemic levels (Office for Health Improvements and Disparities 2024). Alcohol-related harm is estimated to cost the UK National Health Service (NHS) £3.5 billion per year (Scarborough et al. 2011), however this is likely to be underestimated (Institute of Alcohol Studies, 2023). Current UK policy emphasises the importance of improving alcohol care (Public Health England, PHE 2016; Five Year Forward View for Mental Health, Mental Health Taskforce 2016).

One group of people who experience high levels of harms are those who frequently attend hospital in crisis for alcohol-related reasons (Blackwood et al. 2021). Such people often have complex mental and physical health needs and experience unstable housing, and/or social isolation (Neale et al. 2017). Yet traditional service models struggle to adapt to wider social issues that contribute to individuals’ alcohol consumption and capacity to engage in support (Gilburt et al. 2015). Additionally, individuals may face barriers to engaging with specialist addiction treatment services (SATS) due to limited knowledge of existing services, inconvenient service locations, and poor cohesion between healthcare services (Roberts et al. 2020).

Alcohol Assertive Outreach Treatment (AAOT), adapted from assertive outreach treatment in mental health services, is a model that seeks to improve engagement and treatment retention in this population (Blackwood et al. 2020). AAOT involves six essential components (see table 1) including small practitioner-service user caseloads, regular community-based appointments, and assertive and persistent attempts at contact (Drummond et al. 2017). AAOT literature remains in its infancy, yet positive reports of the use of an assertive outreach approach to substance use have been noted in the USA and the Netherlands (Fisk et al. 2006; Roeg et al. 2007). UK Pilot studies have shown that AAOT is a feasible approach to engage individuals with history of difficulty engaging with SATS (Passetti et al. 2008; Drummond et al. 2017), and may result in reductions in hospital admissions, A&E attendances, and service costs (Hughes et al. 2013).

**Table 1:** Six essential components of AAOT (Fincham-Campbell et al. 2018)

A maximum caseload of between 10 and 20 patients per practitioner Input from a multidisciplinary team in form of input from at least three different professions including nurses, medical and psychology or community support and drug workers. Regular contact between patient and practitioner (at least once a week). At least 50% of contacts occurring outside of the service settings, either in patients’ homes or local community settings. A focus on both health and social care needs, including accommodation, finance, leisure, occupation, and physical and mental health. Extended care provided for a prolonged period of 12 months

Despite AAOT being recommended as an intervention for alcohol care by PHE (2016), AAOT is not yet routinely offered within UK alcohol care services (Fincham-Campbell et al. 2018). Where AAOT is implemented, few services provide the six components viewed as essential to high-level care. One survey found only six out of thirty-seven AAOT services in England provided a high-level service comprising five or more key components (Fincham-Campbell et al. 2018). Understanding how high-quality AAOT can be implemented is vital to increasing its uptake and potential impact (Keith et al. 2017). To do this, multilevel influences, such as outer setting external pressures or inner setting culture, which may promote or inhibit implementation over time, must be understood (Damschroder et al. 2022). The Consolidated Framework for Implementation Research (CFIR) is a well-established determinant framework in health science which consists of five overarching domains (the Innovation; Outer Setting; Inner Setting; Individuals; Implementation Process), each with related constructs. CFIR uses these domains and constructs to explain barriers and facilitators to successful implementation and sustainability of interventions (see Damschroder et al. 2022). This study employed the CFIR to analyse data obtained from clinical staff working in AAOT delivery and key stakeholders involved in AAOT service commissioning and policy, to gain an in-depth understanding of determinants influencing implementation of two AAOT services in North West England. In doing so, the study aimed to provide insights that could inform future services hoping to adopt or adapt AAOT in their contexts.

## Methods

A qualitative research study using CFIR-informed semi-structured interviews was conducted in two AAOT services in the Greater Manchester area of North West England, UK.

### Settings of involved AAOT teams

The involved AAOT teams serve growing, diverse communities in Greater Manchester, with high (Service 1, (S1)) to medium (Service 2, (S2)) fidelity to the AAOT model (Fincham-Campbell et al. 2018). S1, established in 2011, supports service users who have presented to the local hospital with alcohol-related difficulties at least twice a month. Therefore, it does not accept referrals but is alerted of individuals who would meet their criteria for support by hospital data analysts. S1 provides assertive recovery support for an average of 12 months or longer. S2, established in 2018, receives referrals from any source and offers shorter-term assertive interventions to promote engagement with the SATS. As the aim of this paper is to explore determinants of the implementation of AAOT, data was analysed together unless specific differences in team aims related to differences in determinants.

### Participants and recruitment

Staff members who delivered care within the two involved AAOT teams were invited to participate in the research, which included outreach workers and team leaders, via email. All invited staff members participated in the research (n = 20). Stakeholders, considered to be individuals who were key informants in health policy, such as service commissioners, national policymakers, clinicians and managers who had developed AAOT teams across the country or researchers in AAOT, were invited to participate in an interview, via email (n = 27). Of those invited, 20 individuals agreed to participate and seven did not respond to the invitation. An additional group of recovery co-ordinators who work in a local SATS were asked to participate in the research. The SATS team manager asked all staff to volunteer, of which eight individuals agreed to participate via email, as key stakeholders, to provide data on the transition between AAOT and SATS. These participants were likely to have a critical understanding of issues surrounding contextual factors that may impact the implementation of AAOT. All individuals were emailed a participant information sheet which emphasised the voluntary nature of their participation and demonstrated their interest in being involved by replying to the email. Interviews were then arranged, and individuals were emailed a consent form to be completed and returned to researchers prior to their interview. As such, written informed consent was gained from all participants.

### Data collection

Semi-structured interviews were completed with n = 20 staff (10 female;10 male) and n = 28 stakeholders (18 female; 10 male) between December 2023 and June 2024. Interviews were conducted over Microsoft Teams or in person, depending on individual circumstances, and were audio-recorded and transcribed verbatim by researchers. Interviews were based on a topic guide based on CFIR criteria. Development of the topic guide was led by the research team and experts by experience advising on wording, structure, and content, prior to ethical approval. Example questions included, ‘Discuss what does AAOT bring in comparison to other interventions’ and ‘Discuss external drivers (e.g. National Policy, local competition) that influenced the decision to implement AAOT’.

### Data analysis

Framework analysis allows for the application of an established framework that is used for informing policy and practice in healthcare settings (Ward et al. 2013). Framework analysis is a form of thematic analysis which applies a framework to identify, describe, and interpret key patterns across cases and themes within the data (Goldsmith 2021). Therefore, interview data were analysed with the Framework Analysis approach (Ritchie and Spencer 1994) using CFIR constructs to create a codebook in NVivo 12. After data familiarisation, deductive coding was carried out on a case-by-case basis based on this codebook. Inductive coding was used to capture original and meaningful themes not included in the CFIR codebook. Two transcripts from each participant group were coded together by two researchers in the first instance, to ensure researchers were aligned in how the framework was being applied to data. All remaining transcripts were coded independently by two researchers. Researchers discussed their coding to ensure consistency in the analysis at regular intervals. Following coding, data within each construct was charted into framework matrices in NVivo 12 and exported to Microsoft Excel. Researchers reviewed the data and wrote interpretative notes for coded quotes. Themes were constructed based on similarities and divergencies in notes which contributed to understanding the barriers and facilitators to the implementation of AAOT.

**Reflexivity** The research team had a background in research and clinical practice in addictions services and lived experience of alcohol addiction and engagement with AAOT. Having this range of experiences of addiction services, including AAOT, was beneficial during interviews to develop rapport with participants and enabled interviewers to probe for further depth in interviewees responses. It also contributed to the data analysis process as researchers could understand the context and meaning of data which supported data interpretation.

## Results

Overall, participants were positive about AAOT, reporting it as acceptable and beneficial for service users. Three main themes (see Table 2) were constructed to highlight key determinants of the successful and sustainable implementation of AAOT.

**Table 2:** Overview of Themes and Associated CFIR Domains

**Themes**	**CFIR Domains**
Organisational and Individual Level Factors: Team Culture and Staff Characteristics	Inner Setting
	Individuals
Systemic Partnerships and Interagency Communication	Outer Setting
	Inner setting
An Adaptable Model Driven by Research and Evaluation	Implementation Process
	Innovation
	Inner Setting

### Organisational and individual level factors: Team culture and staff characteristics

Participants recognised the importance of positive team culture, and well-supported and appropriately skilled staff as resources contributing to the successful implementation and sustainability of AAOT. Interview accounts suggested it was essential that AAOT teams encouraged a learning culture to continue to meet the needs of service users. To create a learning-based environment, participants noted the access to regular formal training opportunities, such as motivational interviewing courses. Additionally, informal opportunities were also valued, where staff members are given time to reflect on and discuss practice through regular team meetings, being given permission to make mistakes, shadowing staff, and feeling able to ask team members questions. Participants valued these learning opportunities as they promoted team problem-solving and development and, in doing so, helped to better meet the varying needs of service users.

*“…sharing when you've done something bold and even if it hasn't worked, sharing that, and giving people permission to make mistakes, that's all right, as long as you're doing things for the right reason, […] it's a learning culture, that's what I think, it's massively important, you need your staff to feel safe to learn…” (STK15)*

Participants also spoke about the necessity of a supportive team environment due to the complexity of the situations AAOT staff manage, often dealing with mortality, high risk, and multiple needs. A team environment that prioritised staff support was highlighted as important for managing staff wellbeing and service user complexities. To create a supportive environment, it was important that staff had access to regular supervision and felt able to ask for help. Having a supportive team environment was also believed to be integral to staff retention which was perceived as contributing to sustainability of AAOT.

“*I think it is sustainable, I think we can sustain it, if you make sure the team are all right, then I think you can go forward, it's about looking after your team and making sure that they are alright and giving them the best chance of going out and helping and supporting these individuals.*” (STA19)

Participants identified the need for staff to have certain qualities, such as being non-judgemental, compassionate, and creative when responding to situations. It also seemed important that staff had the knowledge to identify and respond to physical, mental, and social healthcare needs appropriately. Additionally, participants explained that highly motivated and enthusiastic staff were crucial to AAOT as it inspired hope in service users. Participants also recognised the benefits of having staff from varied personal and professional backgrounds, including those with lived experience. Having a wide range of experiences was felt to ensure there is a breadth of knowledge available within the team, enabling them to problem solve.

*“… in my experience of being here, I’ve had an answer to every question I’ve had to pose from just sharing it in the office, from their experience of doing the job longer than I have, […] there's a pool of experience there, that's massive, from the different sides because what we come across, are commonly recurring themes, homeless, […] involvement with the police, to social services, to children's services, there's experience within the wider team of all those...”* (STA18)

However, participants identified barriers to the sustainability of AAOT. The reduction in the number and variety of professionals was perceived as leading to a less-resourced and efficient service. Additionally, the frequent use of 12-month fixed-term contracts for staff due to funding was perceived as challenging for staff recruitment and retention.

*“…it needs to be appropriately resourced from a workforce of doctors, nurses, psychologists, occupational therapists and recovery coordinators […] healthcare professionals, and what's clear over the last 15 years is, it's just been a race to the bottom”* (STA11)

### Systemic partnerships and interagency communication

Participants identified the importance of having strong partnerships with local GPs, hospitals, commissioners, other local authority services, and other AAOT teams. Service users may experience stigma from other healthcare professionals and services and, consequently, may not receive the support they need. Therefore, participants described the need to have strong relationships with other services so they can advocate for the needs of their service users, through challenging stigma and educating others to improve access to necessary care. Additionally, this can improve attitudes towards funding services such as AAOT teams and enabling service users to access necessary care.

*“…this is not a very popular patient group, you know, and quite often the General Practitioners had enough, the district nurses’ had enough, A&E department has had enough, at this point we get involved and we have to do a lot of advocacy for the patient to try and re-engage the professionals…”* (STK10)

Having trusting partnerships was also perceived as having functional benefits across organisations for service delivery. For example, partnerships with commissioners was perceived as important as successful AAOT was felt to need flexibility in care delivery and data targets, due to the complex needs of service users. Additionally, networks between AAOT teams was highlighted as a way to collectively problem solve patient care issues and develop effective services and pathways. Relationships with acute hospitals were of particular importance as key partnerships for referral pathways to enable service users’ access to AAOT. Participants recognised the requirement to build relationships with other services, for example social services, due to the multiple needs of service users which necessitates a holistic approach. Joint working was perceived to be particularly beneficial for the service user and for the other services understanding the role of AAOT.

*“…it needs to be set up so there's really good multi-agency communication at the system level as well as on the ground at the service delivery level, that seems to be essential because […] people have complex needs they don't just have alcohol needs, they have other needs and working together, that's important”* (STK20)

Participants also spoke about the importance of AAOT teams having close relationships with SATS. There was recognition that the transition to SATS from AAOT can be difficult and anxiety-provoking for service users. Participants felt AAOT teams and SATS should prioritise working together to promote a smooth transition for service users. Whilst some participants reported this happened, others felt joint working could be increased.

*“…diaries are full and we're under a lot of pressure, I think that more emphasis needs to be put on the patient and the individual and how their feelings are, […] the fear, the anxiety, everything that's going on, to meet someone new, to try and share your story, it is traumatic, so if you can put the patient at ease, more progress would be made earlier on […] 20 minutes for an RC [recovery co-ordinator], an outreach worker and the patient being in one room together would put the patient at ease.”* (STK27)

To build partnerships, participants explained clear communication was fundamental and noted how this could best be facilitated. For example, participants explained in-person communication was crucial, which was enabled through close physical proximity of AAOT teams and SATS. Co-located teams were able to talk in person about service users and develop strong relationships. When teams were not based nearby, the relationship appeared to not be as strong. Having access to the same electronic notes system as a form of communication was perceived as being beneficial for AAOT teams to conduct their job as they had access to up-to-date clinical and contact details. Outreach and delivery of training on AAOT to other services was thought to be key to appropriate referrals and access for service users. Participants felt team managers were pivotal in this communication.

*“…the collaborative working and the relationship that you build with the team, they are visible and you get to know them, you build a relationship with them, and I think that is where a lot of the success lies, I can ring them up and go ‘I just wanna check’, […] I think that works very well as opposed to […] you're not sitting behind the emails.”* (STK27)

*“…part of it is going out and engaging with other organisations and doing, delivering training sessions to staff and volunteers and sometimes service users to let them know what our role is, how we fit into the service, what we can and what we can't do…”* (STA16)

Participants reported barriers to developing strong partnerships. For example, participants described difficulties building relationships with mental health services who typically rejected referrals for individuals with alcohol dependence and had limited capacity to joint work. Other barriers included when other services did not understand the role of AAOT staff and made referrals and requests for work that was not within the scope of an AAOT worker, and in some cases, undermined their role.

*“…some of them will ask you to do things that don't aren't in your job criteria, they're really not in your job criteria, they can quite easily do themselves, and then that can make you feel as though someone's treating you as though you’re their personal assistant…”* (STA09)

### An adaptable model driven by research and evaluation

Adaptability of the AAOT model, whilst maintaining fidelity, was identified as being critical to its implementation and service delivery, to continue to meet service user needs. Participants highlighted the importance of research and evaluation to understand local service user need to drive service adaptations. For example, adaptations in how service users are identified, or the length of treatment offered. Participants felt that based on the needs of local service users, AAOT teams should be flexible and for those who are unable to engage with SATS, longer-term treatment within the AAOT team should be offered.

*“We was reviewing the data every six months and quite often you'd miss the boat with certain clients and it wasn't as effective as what we wanted, so then we started looking at the two alcohol related admissions within a month period, we set up a system through the hospital where we would get alerted when people were coming into hospital so we would get an email alert so we could monitor people quite closely and quite quickly, so that just sped everything up.”* (STA03)

*“…a helpful model would be ideally one that could incorporate both those poles if you like, that where it's possible, bring people into the mainstream service after a period of time […] however, if people are just not able to do that, […] there should be an element of the service that could continue to put work with people in an ongoing way”* (STK20)

Additionally, participants discussed the need for research and evaluation to demonstrate the impact and effectiveness of AAOT teams. This can be reported to stakeholders and decision makers to support the acquirement of funding, which is integral to sustainability. AAOT leaders should consult with relevant stakeholders to identify optimal impact metrics for reporting purposes. Subsequently, they should establish robust data collection mechanisms to ensure the systematic reporting of service user outcomes.

*“…make sure that you're collecting data that shows that your service model is working and that you're reaching the right people and that it's actually having an impact and therefore you can build a case to, to continue the funding […] so investing some resource in collecting routine clinical data and outcome data, actually is beneficial to the sustainability”* (STK10)

## Discussion

Despite positive evidence for high quality AAOT as an acceptable, feasible and effective model of care for people who frequently attend hospital in crisis for alcohol-related reasons, implementation remains sub-optimal (Fincham-Campbell et al. 2018). This qualitative study used CFIR-informed interviews to explore the views and experiences of 48 frontline practitioners and other stakeholders involved in two AAOT services based in North West England in order to better understand which factors appear to shape successful implementation and sustainability. Three themes based on the CFIR framework were identified: organisational and individual level factors: team culture and staff characteristics; systemic partnerships and interagency communication; and an adaptable model driven by research and evaluation.

Factors at an organisational and individual practitioner level were perceived as key influences on the implementation and sustainability of AAOT. At an organisational level, staff were recognised as an essential resource to AAOT, so a culture which prioritised continued staff development and support was integral to both meeting service user needs and staff retention, and ultimately the sustainability of AAOT teams. Findings support previous research which also highlights the importance of an environment which encourages learning and new ideas for addiction service implementation (Simpson et al. 2007; Becan et al. 2012) and research which emphasises the role of supervision for staff retention and ultimately, improved outcomes for service users (Van De Ven et al. 2020). Existing research regarding the role of practitioner attitudes and characteristics in influencing implementation is unclear (Louie et al. 2022), and so this study’s findings can offer some clarity. Specifically, individual practitioner level factors were perceived as being crucial, such as feeling capable, being motivated, and having a range of professional and personal experiences within the team to meet the needs of service users. Endorsing a team culture which prioritises learning and support in these ways can enable psychological safety and improve team performance (O’Donovan and McAuliffe 2020).

At a systemic level, partnerships between organisations were key influencers in AAOT implementation and sustainability. Particularly, partnerships between AAOT teams and commissioners, other AAOT teams, and other health and social care services were described as important for enabling service users to access AAOT, managing varying needs of service users, and supporting transitions between services. This supports previous research which highlighted the importance of inter-agency relationships in influencing referral pathways and therefore access to healthcare services (Sword et al. 2013). To develop strong partnerships, in-person communication was vital, with services being based nearby leading to stronger relationships and communication relating to service users, echoing previous findings (Corbin et al. 2016; Savic et al. 2017). This also supports previous research demonstrating the importance of clear communication from managers to support the development of partnerships between services (Louie et al. 2022). This research extends the literature and demonstrates the importance of AAOT teams doing training sessions with other services to challenge stigma and increase the visibility and understanding of the role of AAOT teams, which can strengthen partnerships. Prioritising interagency partnerships aligns with the recent implementation of Integrated Care Systems, where partnerships across services and organisations are emphasised to meet individual needs (Department of Health and Social Care 2024).

Research was identified as a crucial resource for demonstrating the need and impact of AAOT to secure further funding which ultimately determines sustainability of AAOT teams. However, AAOT teams may not routinely have easy access to data or the skills to conduct evaluations. This can negatively impact funding requests and any adaptations needed for the model. It may be that healthcare services need to adopt a more robust approach to evaluations of practice, dedicating sufficient resources to service evaluation and quality improvement. This supports a recommendation in the Dame Carol Black review, which emphasises the importance of outlining the effectiveness of alcohol interventions (Department of Health and Social Care 2021).

Implementation research in alcohol services is sparse. In the research that does exist, multilevel influences on implementation are neglected and, to the authors' knowledge, there is no implementation research involving AAOT. This research has, therefore, begun to fill two gaps in the literature. Firstly, it has responded to calls for prioritising implementation research in addictions services and demonstrated the importance of multilevel influences in implementation, for example, from within the organisation (team culture, staff factors), external influences (partnerships), and stages of the implementation process (reflecting and evaluating) (Louie et al. 2021). Secondly, it has conducted this research in an under-researched area and given insight into factors influencing implementation of AAOT. Such research is necessary for improving uptake and effectiveness of AAOT and to improve the wellbeing of individuals who experience alcohol dependence.

## Limitations

It is important to note that the findings relate to the two involved AAOT teams based in North West England. However, the services had sufficient fidelity to the AAOT model and recruited participants from across England which supports the generalisability of the results and recommendations. Selection bias was avoided at the staff level by interviewing all members of the two teams. At the key stakeholder level, although a wide range of opinions were sought, selection bias cannot be ruled out, due to purposeful sampling. However, social desirability bias may have changed the participants’ answers due to the potential commissioning implications of the research, despite data being anonymised. To overcome such issues, participants were encouraged to think critically to aid service development. Additionally, this study only explores the perspectives of professionals and not service users. Future research is encouraged to focus on service user perspectives.

## Conclusion and recommendations

The AAOT model was highly regarded by the staff and stakeholders involved in its implementation and delivery. Key themes were ‘organisational and individual level factors: team culture and staff characteristics’; ‘systemic partnerships and interagency and communication’; and ‘an adaptable model driven by research and evaluation’. Individuals involved in the implementation of AAOT should consider the following recommendations:

- Create a team culture which prioritises staff support, learning, and development. Ensure there are opportunities for training, case discussions, and supervision.
- Build partnerships with other agencies based on clear in-person communication to share the role of AAOT and develop referral pathways.
- Understand the needs of the locality and local service users and adapt the implementation model accordingly.
- Use evaluation to demonstrate the need, impact and adaptability of AAOT teams to secure funding and sustainability.

## Supplementary Material

VALOR_STAFF_TOPIC_GUIDE_v_2_03_10_2023_agaf003

VALOR_STAKEHOLDERS_TOPIC_GUIDE_v_2_03_0_2023_agaf003

## Data Availability

The data underlying this article cannot be shared publicly for the privacy of individuals that participated in the study.
