# Supplementary material for: A qualitative exploration of the barriers and facilitators to the implementation of the alcohol assertive outreach model
Source: Alcohol Alcohol. 2025 Feb 3;60(2):agaf003. doi: 10.1093/alcalc/agaf003 (PMC11788419; doi:10.1093/alcalc/agaf003)
Supplement: VALOR_STAFF_TOPIC_GUIDE_v_2_03_10_2023_agaf003 [file valor_staff_topic_guide_v_2_03_10_2023_agaf003.docx]

**VALOR Study - Staff Topic Guide for Semi-Structured Interview**

**Preamble:**

Reminder of the purpose of study

Explanation of ethics, consent and confidentiality of interview and analysis

Structure of the interview (may be some overlap in questions and responses)

**Introduction / Orientation:**

The purpose of this study is to evaluate the alcohol assertive outreach model, find out when and who it works well for, and what we can do to improve it.

Alcohol Assertive Outreach Teams offer support to people with alcohol use problems that have previously struggled to engage with structured alcohol care (i.e., community alcohol teams). This group might have complex needs (for e.g., disabilities, housing, or employment issues) that act as barriers to engagement, and as a result have more emergency healthcare utilisation (for e.g., A&E or inpatient care). AAOT services aim to overcome these barriers by using assertive and flexible strategies, and by offering appointments and resources within the community. The six core components of AAOT are:

1. Small caseloads per practitioner
2. Input from a multidisciplinary team in form of contributions from at least three different professions including nurses, medical and psychology or community support and drug workers.
3. Regular contact between you and practitioner (at least once a week).
4. At least 50% of contacts occurring outside of the service settings, either in your home or local community settings.
5. A focus on both health and social care needs, including accommodation, finance, leisure, occupation, and physical and mental health.
6. Extended care provided for a prolonged period of 12 months

You have been invited to interview because you are a member of staff involved in AAOT services, to share your experience and insight on the assertive outreach model and how it works in practice. We will ask you about the barriers and facilitators to the delivering care within the AAOT model and use this to inform our suggestions for best implementation practice.

**TOPIC GUIDE**

1. **Individual understanding and experience of AAOT**

Prompts:

- To describe their role in the implementation of AAOT model and how long they have been working in this role
- Explore/remind interviewees the different components of AAOT model (whether they/their service delivered all of them, importance, interaction between components)
- Characteristics of service users (age, ethnicity, housing, mental health)

**Exploring impact of AAOT**

Prompts:

- Methods of evaluating the outcomes of AAOT as a team and as individual key workers
- How would they measure/assess the impact of AAOT (e.g. number of contacts, service user alcohol misuse, improvement of living circumstances)
- Whether aspects of their approach changed along the way to accommodate service user and staff needs
- Advantages and disadvantages of the AAOT model for service users (person-centred care, patient outcomes, engagement with other structured or emergency services)
- Advantages and disadvantages of the AAOT model for staff (lone working, smaller caseloads, professional development)
- Whether it makes a difference in reducing the costs of healthcare services

1. **Barriers and facilitators to the uptake and implementation of the AAOT model**

Prompts:

- Things that helped (e.g. for individuals, within and between organizations)
- Barriers/challenges in working in AAOT (risk to staff, complex cases, ways to manage or overcome these challenges)
- necessary personal characteristics for an AAOT keyworker
- Why certain service users turn down the support offered by AAOT

1. **Understanding and experiences of AAOT implementation across organisations**

Prompts:

- Whether organisations involved in AAOT share a common understanding of the model and the method of its implementation
- Relationship between the different services involved
- Communication and interaction between service providers
- Organisational characteristics for an effective implementation of AAOT (flexibility, size, experience)
- Promotion and visibility of AAOT across the service footprint
- Things that work well and things that do not

1. **Sustainability of changes driven by the AAOT**

Prompts:

- How sustainable is AAOT in the long term
- Resources needed to make AAOT sustainable
- Actions needed to make AAOT sustainable
- Challenges to the sustainability of AAOT

1. **Anything else the participant wants to add**

-Thank participant and end the interview-
