# Supplementary material for: A qualitative exploration of the barriers and facilitators to the implementation of the alcohol assertive outreach model
Source: Alcohol Alcohol. 2025 Feb 3;60(2):agaf003. doi: 10.1093/alcalc/agaf003 (PMC11788419; doi:10.1093/alcalc/agaf003)
Supplement: VALOR_STAKEHOLDERS_TOPIC_GUIDE_v_2_03_0_2023_agaf003 [file valor_stakeholders_topic_guide_v_2_03_0_2023_agaf003.docx]

**VALOR Study – STAKEHOLDERS Topic Guide for Semi-Structured Interview**

**Preamble**

Reminder of the purpose of study

Explanation of ethics, consent and confidentiality of interview and analysis

Structure of the interview (may be some overlap in questions and responses)

**Introduction**

The purpose of this study is to evaluate the alcohol assertive outreach model, find out when and who it works well for, and what we can do to improve it.

Alcohol Assertive Outreach Teams offer support to people with alcohol use problems that have previously struggled to engage with structured forms of alcohol care (e.g., alcohol care teams). This group often has complex needs (for e.g., disabilities, housing, and employment issues) that might act as barriers to engagement, and consequently have more emergency healthcare utilisation (A&E or inpatient care). AAOT services aim to overcome these barriers by using assertive and flexible strategies and offering appointments and resources within the community. The six core components of AAOT (Fincham-Campbell et al, 2018) are:

1. Small caseloads per practitioner
2. Input from a multidisciplinary team with contributions from at least three different professions including nurses, medical and psychology or community support and drug workers.
3. Regular contact between you and practitioner (at least once a week).
4. At least 50% of contacts occurring outside of the service settings, either in your home or local community settings.
5. A focus on both health and social care needs, including accommodation, finance, leisure, occupation, and physical and mental health.
6. Extended care provided for a prolonged period of up to 12 months

You have been invited to interview because you are a key stakeholder for AAOT services, which means you might be involved in local policy, commissioning, or developing guidelines that impact AAOT services. We will ask you to share your experience and insight regarding the barriers and facilitators to implementing this approach, and use this to inform suggestions for best practice, including adaptability and sustainability of the AAOT model.

**TOPIC GUIDE**

1. **Exploring individual involvement with the implementation of AAOT**

Prompts:

- Describe their role in the implementation of the AAOT model
- Explore/remind interviewees the different components of AAOT model (whether they/their service delivered all of them, importance, interaction between components)
- Steps/decisions they made in setting up the AAOT

1. **Innovation**

Prompts:

- Discuss what does AAOT bring in comparison to other interventions
- The environment and the needs identified in the local context
- Appropriateness
- Training needs

1. **Outcomes**

Prompts:

- Their involvement in evaluating the implementation of AAOT
- Methods of evaluation of the service (data or general perception)
- Reduction of healthcare costs (A&E attendances, reduced mortality)
- AAOT as an example of a person-centred approach
- Outcomes for service users
- Outcomes for staff

1. **Understanding and experiences of AAOT implementation across organisations**

Prompts:

- External drivers (e.g. National Policy, local competition) that influenced the decision to implement AAOT

Aspects of organizational culture that affect how AAOT is implemented and delivered (client-centred approach, flexibility, size)

Resources necessary to successfully implement AAOT

- Perspectives of other key influencers in the healthcare system about AAOT/ champions of the AAOT model
- Promotion and visibility of AAOT across their service footprint

1. **Barriers and facilitators to the implementation of the AAOT model**

Prompts:

- Factors that facilitate the implementation of the AAOT model

(local commissioning, national policy groups/national stakeholders)

- Barriers or challenges to implementing AAOT/ways to manage or overcome these challenges
- Reasons why the model is not adopted widely

1. **Sustainability of the AAOT**

Prompts:

- How sustainable is AAOT in the long term
- Resources/actions needed to make the AAOT sustainable
- Challenges to the sustainability of AAOT as an approach

**7. Anything else the participant wants to add**

-Thank participant and end the interview-
